# Proof-of-concept for characterization of neurodegenerative disorders utilizing two non-REM sleep biomarkers

**DOI:** 10.3389/fneur.2023.1272369

**Published:** 2023-10-20

**Authors:** Daniel J. Levendowski, Thomas C. Neylan, Christine M. Walsh, Debby Tsuang, David Salat, Joanne M. Hamilton, Joyce K. Lee-Iannotti, Chris Berka, Gandis Mazeika, Bradley F. Boeve, Erik K. St. Louis

**Affiliations:** ^1^Advanced Brain Monitoring, Inc., Carlsbad, CA, United States; ^2^UCSF Weill Institute for Neurosciences, University of California, San Francisco, San Francisco, CA, United States; ^3^Memory and Aging Center, University of California, San Francisco, San Francisco, CA, United States; ^4^Geriatric Research Education and Clinical Center, VA Puget Sound Health Care System, Seattle, WA, United States; ^5^Massachusetts General Hospital, Charlestown, MA, United States; ^6^Advanced Neurobehavioral Health, San Diego, CA, United States; ^7^Banner University Medical Center, Phoenix, AZ, United States; ^8^Department of Neurology and Center for Sleep Medicine, Mayo Clinic College of Medicine and Science, Rochester, MN, United States

**Keywords:** sleep spindles, non-REM hypertonia, neurodegenerative disease, sleep biomarkers, Alzheimer’s disease, parkinsonism

## Abstract

**Study objective:**

This proof-of-concept study aimed to determine whether the combined features of two non-rapid eye movement (NREM) sleep biomarkers acquired predominantly in-home could characterize different neurodegenerative disorders.

**Methods:**

Sleep spindle duration and non-REM hypertonia (NRH) were evaluated in seven groups including a control group (CG = 61), and participants with isolated REM sleep behavior disorder (iRBD = 19), mild cognitive impairment (MCI = 41), Parkinson disease (PD = 16), Alzheimer disease dementia (ADem = 29), dementia with Lewy Bodies or Parkinson disease dementia (LBD = 19) and progressive supranuclear palsy (PSP = 13). One-way analysis of variance (ANOVA), Mann–Whitney *U*, intra-class (ICC) and Spearman ranked correlations, Bland–Altman plots and Kappa scores, Chi-square and Fisher exact probability test, and multiple-logistic regression were focused primarily on spindle duration and NRH and the frequencies assigned to the four normal/abnormal spindle duration/NRH combinations.

**Results:**

ANOVA identified group differences in age, sleep efficiency, REM, NRH (*p* < 0.0001) and sleep time (*p* = 0.015), Spindle duration and NRH each demonstrated good night-to-night reliabilities (ICC = 0.95 and 0.75, Kappa = 0.93 and 0.66, respectively) and together exhibited an association in the PD and LBD groups only (*p* < 0.01). Abnormal spindle duration was greater in records of PSP (85%) and LBD (84%) patients compared to CG, MCI, PD and ADem (*p* < 0.025). Abnormal NRH was greater in PSP = 92%, LBD = 79%, and iRBD = 74% compared to MCI = 32%, ADem = 17%, and CG = 16% (*p* < 0.005).The combination biomarker normal spindle duration/normal NRH was observed most frequently in CG (56%) and MCI (41%). ADem most frequently demonstrated normal spindle duration/normal NRH (45%) and abnormal spindle duration/normal NRH (38%). Normal spindle duration/abnormal NRH was greatest in iRBD = 47%, while abnormal spindle duration/abnormal NRH was predominant in PSP = 85% and LBD = 74%.

**Conclusion:**

The NREM sleep biomarkers spindle duration and NRH may be useful in distinguishing patients with different neurodegenerative disorders. Larger prospective cohort studies are needed to determine whether spindle duration and NRH can be combined for prodromal assessment and/or monitoring disease progression.

## 1. Introduction

Sleep impacts brain health by enabling glymphatic clearance, thereby reducing possible toxic metabolites that accumulate during wakefulness (1), and facilitating synaptic homeostasis and consolidation (2, 3). Conversely, insufficient sleep may result in buildup of beta amyloid, tau, and synuclein proteins, and compromised cognitive function (e.g., memory and learning) (4–7).

Studies suggest biomarkers measured during sleep hold promise in the characterization and monitoring of neurodegeneration along the continuum of prodromal disease, symptomatic mild cognitive impairment and eventual dementia. Decreased sleep spindle oscillations have been associated with cognitive decline in older adults, increased tau levels, and development of dementia in patients with Parkinson disease (8). Patients with Alzheimer disease dementia and progressive supranuclear palsy also exhibit reduced spindle activity, reflecting decreased thalamocortical network neuronal activity (9). Amongst individuals with cognitive decline, those with increased muscle activity during rapid eye movement (REM) sleep, known as REM sleep without atonia (RSWA), most often have synucleinopathy pathology (10–14). In our pilot study, sleep spindle duration and non-REM hypertonia (NRH), a recently validated sleep biomarker, were each independently associated with a range of Parkinsonian spectrum disorders (15).

The aim of this proof-of-concept study was to determine whether features of the non-REM (NREM) sleep biomarkers spindle duration and NRH could be combined to characterize patients associated with specific neurodegenerative disorders. Biomarkers found to be orthogonal and predictive make good candidates for machine learning algorithms that might distinguish patients with prodromal or early stage disorders [i.e., isolated rapid eye movement sleep behavior disorder (iRBD) and mild cognitive impairment (MCI)], from those with the manifest neurodegenerative disorders [i.e., Parkinson disease (PD), Alzheimer disease dementia (ADD), dementia with Lewy bodies (DLB), Parkinson disease dementia (PDD), and progressive supranuclear palsy (PSP)]. This study foreshadows eventual application of a comprehensive sleep neurodegenerative profiling capability that could be acquired in the patient’s home.

## 2. Methods

### 2.1. Participants

Seven groups totaling 194 records were acquired under IRB review and approval at each of the seven study sites, including 61 controls without known cognitive impairment (CG = control group with mini-mental state exam score ≥ 29), 19 patients with iRBD, 41 with MCI, 16 with PD, 29 with ADem, 19 with Lewy body dementia (LBD: DLB = 15, PDD = 4), and 13 with PSP. A detailed description of the criteria used for selection of CG, MCI, PD, ADem, LBD, and PSP participants was previously described (15). iRBD participants were diagnosed according to the International Classification of Sleep Disorders, 3rd edition diagnostic criteria, which required the presence of RSWA documented by polysomnography (PSG) and dream enactment behavior based on a clinical history or video-PSG recording (16).

### 2.2. Recordings

Sleep Profiler (SP) recordings were acquired from electroencephalography (EEG) sensor sites AF7-AF8, AF7-Fpz and AF8-Fpz (Advanced Brain Monitoring, Carlsbad, CA, USA) with self-application in the participants home, or during simultaneous in-lab PSG acquisition in iRBD patients. The SP records were auto-staged using previously described machine-learning algorithms intended to conform to the standard American Academy of Sleep Medicine guidelines for the visual characterization of sleep (17, 18). The artificial intelligence-based staging algorithms incorporated within-epoch temporal power spectral characteristics in combination with auto-detected individual slow waves, sleep spindles, cortical arousals, and electromyographic (EMG) excursions, and the within-epoch phasic correlations across the two signals containing electrooculography activity (i.e., AF7-Fpz versus AF8-Fpz). The auto-staging accuracy with and without technical review was validated compared to visual scoring of PSG records in subjects including those referred for an assessment of sleep disordered breathing (17). Staging accuracy in patients with iRBD (subsequent to technical edits based on SP Guidelines for Patients with Neurodegenerative Diseases) was also validated in comparison to simultaneously acquired PSG (19, 20).

### 2.3. Sleep spindles

**Figure 1:**
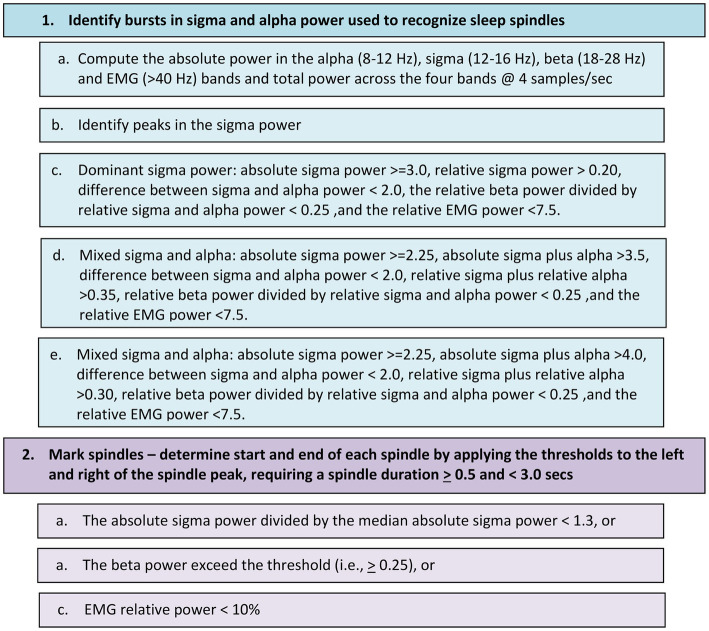
Flow chart for detecting sleep spindles in the EEG signal differentially recorded from the frontopolar sites Af7 and Af8.

Automated sleep spindle detection began with recognition of an excursion in sigma power (12–16 Hz) in combination with burst of alpha (8–12 Hz) power measured in 250 millisecond increments (Figure 1). Combinations of sigma and alpha thresholds ensured both fast and slow spindles were detected, irrespective of amplitude. Additional thresholds were applied to the beta and EMG bands to reduce the incidences of medication-related false-positive events. Sleep spindles were required to have a minimum duration of 0.5-s, a maximum duration of 3-s, and to be non-overlapping with cortical arousals.

Spindle duration was computed as the sum of the elapsed times for all sleep spindles during N2 and N3 sleep. Spindle density was based on the number of spindles divided by the total minutes of stages N2 and N3. A threshold for abnormal spindle duration was set at 1 min or less based on receiver operating characteristic (ROC) curves comparing the ADem, LBD/DLB and PSP groups to the CG, iRBD, MCI, and PD groups in this cohort. This optimized threshold yielded an area under the curve (AUC) of 0.65 (95% confidence interval range 0.57–0.72), with sensitivity of 0.66 and specificity of 0.64. This threshold was then used to differentiate abnormal/normal spindle density for orthogonal classifications within and across the groups.

### 2.4. Non-REM hypertonia (NRH)

Factors leading to its discovery and a flow chart detailing the criteria employed for the automated detection of NRH were recently described (15). The algorithms included pattern recognition of persistently elevated electromyographic (EMG) power relative to delta, theta, and sigma bands within each 30-s epoch. Variability thresholds were applied within and across a minimum of four contiguous epochs to ensure EMG bursts attributed to sleep-disordered breathing arousals, movements, etc. were not mischaracterized as NRH. Rules were then applied to combine epochs into contiguous epoch blocks of NRH.

NRH was calculated as a percentage of total sleep time based solely on auto-detected blocks, i.e., no edits were made to add or remove NRH. Incidences of NRH were excluded during the first 10 min of elapsed sleep at onset or 10 or more minutes of upright time. The threshold for abnormal NRH was determined to be ≥5% of sleep time using ROC analysis yielding an AUC of 0.77, and having sensitivity of 0.75 across iRBD, PD, PDD, DLB and PSP, and specificity of 0.79 for CG, MCI and ADem, equivalent to a previous published similar analysis from the NRH validation study which did not include iRBD patients (15).

### 2.5. Data analysis

Inter-class correlations (ICC), Bland–Altman plots (bias ±1 standard deviation), and Kappa scores were used to assess night-to-night variability in the 147 in-home SP recordings records with two-nights of data (i.e., 76% of all recordings). The sleep metrics for two-night studies were then weight-averaged [i.e., Night 1 value × (sleep times night 1/ nights 1 + 2) + Night 2 value × (sleep times night 2/ nights 1 + 2)]. One-way Analysis of variance (ANOVA) was used to identify variables that characterized group differences. Spearman ranked correlations were used to assess the interrelationships between spindle durations and NRH for each group. Mann–Whitney *U* tests were used to identify pairwise group differences in demographic, sleep, medications and NREM biomarker characteristics. Multiple logistic regression models were used to assess factors that influence spindle duration and NRH using all records except one PSP and three PD patients with missing medication information. The “dopaminergic agents” category included both dopamine agonists and levodopa formulations, while the “AChE/NMDA” category included both acetylcholinesterase inhibitors and N-Methyl D-aspartate receptor antagonists.

Tallies were made, group distributions were computed, and the NREM biomarkers were combined into four different categories based on the pairing of abnormal/normal spindle duration and abnormal/normal NRH. For individual variable pairwise comparisons, statistical significance was set at *p* ≤ 0.025 using a Bonferroni correction. For the multiple logistic regression and orthogonal variable combinations, significance was set to alpha level of 0.05. Chi-square and Fisher exact probability tests were used to compute the odds ratios (OR) related to differences in the distributions of cases assigned to each orthogonal category. All results are presented in the same group order (i.e., CG, iRBD, MCI, PD, ADem, LBD, PSP). Distributions of sleep metrics and between-group comparisons of abnormal NRH for the CG, PD and PSP groups were previously reported (15).

## 3. Results

### 3.1. Across group comparisons

One-way ANOVA was used to evaluate group effects on the demographic, sleep, and NDD biomarker variables presented in Table 1. Group differences were most pronounced in age, sleep efficiency, %REM, and NRH (all *p* < 0.0001), followed by sleep time (*p* = 0.015). Spindle duration (*p* = 0.052) and spindle density (*p* = 0.065) both trended toward significant group differences.

**Table 1:** Distributions of key demographic, sleep, medications characteristics with greater or reduced mean ± SD values identified.

Group	ANOVA: F (p)	CG	iRBD	MCI	PD	ADem	LBD	PSP
Demographic characteristics								
Number subjects		61	19	41	16	29	19	13
Females, %		49.2	26.3	36.6	31.3	20.7	10.5	53.8
Age, years	6.48 (<0.0001)	64.9 ± 8.7	63.7 ± 9.6	70.7 ± 8.3	67.3 ± 8.7	74.7 ± 6.7	70.0 ± 6.3	69.7 ± 9.0
Conventional sleep metrics								
Sleep time, h	2.72 (0.015)	6.3 ± 0.9	5.6 ± 1.4	6.4 ± 1.3	6.1 ± 1.1	6.4 ± 1.2	5.9 ± 1.9	5.2 ± 1.5
Sleep efficiency, %	11.28 (<0.0001)	86.4 ± 6.7	76.8 ± 16.2	82.3 ± 11.5	79.9 ± 11.6	77.6 ± 17.1	70.6 ± 16.8	58.4 ± 14.1
Stage REM, %	12.63 (<0.0001)	21.2 ± 5.8	20.6 ± 10.0	18.3 + 8.3	18.6 ± 7.6	15.9 ± 6.3	8.0 ± 7.8	7.6 ± 7.6
N1	1.49 (0.18)	6.6 ± 4.2	9.3 ± 5.5	7.0 ± 4.9	6.9 ± 5.1	9.4 ± 9.7	6.0 ± 4.3	10.0 ± 12.2
N2	1.05 (0.39)	49.6 ± 14.4	48.2 ± 15.5	52.5 ± 18.3	49.4 ± 20.8	57.1 ± 19.3	52.2 ± 20.0	58.0 ± 21.6
N3	1.72 (0.12)	21.4 ± 20.8	8.7 ± 7.6	18.4 ± 17.0	14.0 ± 16.2	14.7 ± 21.9	18.2 ± 20.7	9.8 ± 12.1
NREM NDD biomarkers								
Spindle duration, min	2.13 (0.052)	7.9 ± 11.0	3.2 ± 3.4	5.5 ± 9.2	4.6 ± 6.3	3.4 ± 6.3	3.4 ± 8.8	0.9 ± 2.1
Spindle density, events/m	2.02 (0.065)	1.71 ± 2.13	0.90 ± 0.69	1.23 + 1.69	1.14 ± 1.52	0.66 ± 0.99	0.91 ± 2.27	0.40 ± 1.01
Stage NRH, %	14.14 (<0.0001)	3.1 ± 5.1	13.2 ± 12.6	3.8 ± 4.8	11.1 ± 10.5	2.9 ± 3.9	15.7 ± 13.6	15.3 ± 8.8
Medications**								
SSRI/SNRI, %		9.8	47.4	36.6	23.1	31.0	47.4	58.3
Dopaminergic agents, %		0.0	5.3	4.9	61.5	6.9	31.6	50.0
AChE/NMDA, %		0.0	0.0	17.1	7.7	37.9	57.9	8.3
Benzodiazepine, %		0.0	21.1	0.0	30.8	0.0	10.5	0.0

** Denominator adjusted for missing medication information in one PSP and three PD patients.

### 3.2. By group comparisons

There was a greater proportion of women than men in the CG compared to the ADem and LBD groups and in PSP versus LBD (all *p* < 0.025). The CG was younger than MCI, ADem and LBD (*p* < 0.01), iRBD patients were younger than MCI, ADem, and LBD (*p* < 0.025), and PD was younger than ADem (*p* < 0.01).

The CG had greater sleep time compared to iRBD and PSP (*p* ≤ 0.025). The sleep efficiencies of PSP were less than in all other groups (*p* < 0.005), LBD were less than CG and MCI (*p* < 0.002), and the ADem patients were less than CG (*p* < 0.0005). Both the PSP and LBD groups exhibited less REM sleep compared to CG, MCI, PD and ADem (*p* < 0.002), and the ADem had less REM sleep compared to CG (*p* < 0.0005). Spindle duration was reduced in both the PSP and LBD groups compared to CG, iRBD, MCI, and ADem (*p* < 0.025).

The CG had less SSRI/SNRI use compared to iRBD, MCI, LBD, PSP (*p* < 0.001), and ADem groups (*p* < 0.025). Use of dopaminergic agents was greater in PD and PSP patients compared to CG, iRBD, MCI, and ADem (*p* < 0.01), and when the LBD group was compared to CG and MCI (*p* < 0.01). AChE/NMDA use was greater in LBD compared to CG, iRBD, MCI, PD, and PSP (*p* < 0.01), the ADem group versus CG, iRBD and PD (*p* < 0.007), and in MCI patients compared to CG (*p* < 0.01). Benzodiazepine use was greater in the PD versus the CG, MCI, and ADem, and in iRBD patients compared to CG (*p* < 0.002).

### 3.3. Night-to-night reliability of NREM biomarkers

Spindle duration exhibited strong night-to-night reliability (ICC = 0.95, *p* < 0.0001, Bland Altman bias: 0.15 ± 2.66 min). In the two-night recordings, normal or abnormal spindle duration was classified consistently across nights in 97% of records (kappa = 0.93) with 53% being abnormal.

NRH demonstrated moderate night-to-night reliability (ICC = 0.75, *p* < 0.0001, Bland Alman bias: 0.60 ± 6.74%). Normal/abnormal night-to-night consistency was observed in 84% of the records (kappa = 0.66), with 32% characterized with abnormal NRH.

### 3.4. Interrelationships between NREM biomarkers

An association between spindle duration and NRH was observed in PD (*r*_s_ = 0.63, *p* < 0.01) and LBD (*r*_s_ = 0.62, *p* < 0.005) but not in AD (*r*_s_ = 0.02, *p* = 0.91), MCI (*r*_s_ = 0.2, *p* = 0.88). iRBD (*r*_s_ = 0.32, *p* = 0.19) and CG (*r*_s_ = 0.21, *p* = 0.10).

### 3.5. Distributions and factors that influence NREM biomarker abnormality

Abnormal spindle duration was significantly greater for LBD (84%) and PSP (85%) compared to CG (36%), iRBD (32%), MCI (39%), PD (38%), and ADem (45%) (*p* < 0.025). Abnormal NRH was observed in a greater number of PSP patients (92%) versus CG (16%), MCI (32%), and ADem (17%) (all *p* < 0.0005), when LBD (79%) and iRBD (74%) were compared to CG, MCI, and ADem (*p* < 0.005), and for PD (56%) versus CG and ADem (all *p* < 0.01).

Multiple logistic regression models were fit to explore relationships between dependent variables of normal versus abnormal classifications for spindle duration or NRH, each with independent variables sex, age, use of SSRI/SNRIs, dopaminergic agents, AChE/NMDA medications, and/or benzodiazepines, and diagnostic group. Spindle duration was associated with sex (*p* < 0.002; Odds Ratio: OR = 0.34, 95% CI: 0.18–0.67), age (*p* = 0.019; OR = 1.04, 95% CI: 1.007–1.08), benzodiazepine use (*p* = 0.035; OR = 0.15, 95% CI: 0.02–0.87) and diagnostic group (*p* = 0.01; OR = 0.73, 95% CI: 0.57–0.93). NRH was associated with SSRI/SNRI use (*p* < 0.0005; OR = 3.63, 95% CI: 1.81–7.28) but not diagnostic group (*p* = 0.081, OR = 0.82, 95% CI: 0.66–1.02). Table 2 presents the by-group proportions of cases with benzodiazepine use and/or having abnormal spindle duration and cases with SSRI/SNRI use and/or having abnormal NRH.

**Table 2:** Distributions of medication use and/or abnormal spindle duration (SpD) and non-REM hypertonia (NRH) by group.

	Benzodiazepine and spindle duration				SSRI/SNRI and NRH			
Medication use	No	Yes	No	Yes	No	Yes	No	Yes
Abnormal biomarker	No	No	Yes	Yes	No	No	Yes	Yes
NC	57.4%	0.0%	42.6%	0.0%	75.4%	8.2%	14.8%	1.6%
MCI	53.7%	0.0%	46.3%	0.0%	46.3%	22.0%	17.1%	14.6%
PD	38.5%	15.4%	38.4%	7.7%	53.8%	0.0%	23.1%	23.1%
iRBD	31.6%	21.0%	47.4%	0.0%	21.0%	5.3%	31.6%	42.1%
ADem	41.4%	0.0%	58.6%	0.0%	58.6%	24.1%	10.4%	6.9%
LBD	10.5%	5.3%	78.9%	5.3%	21.0%	0.0%	31.6%	47.4%
PSP	16.7%	0.0%	83.3%	0.0%	0.0%	8.3%	41.7%	50.0%

### 3.6. Within-group distributions across NREM biomarker combinations

**Figure 2:**
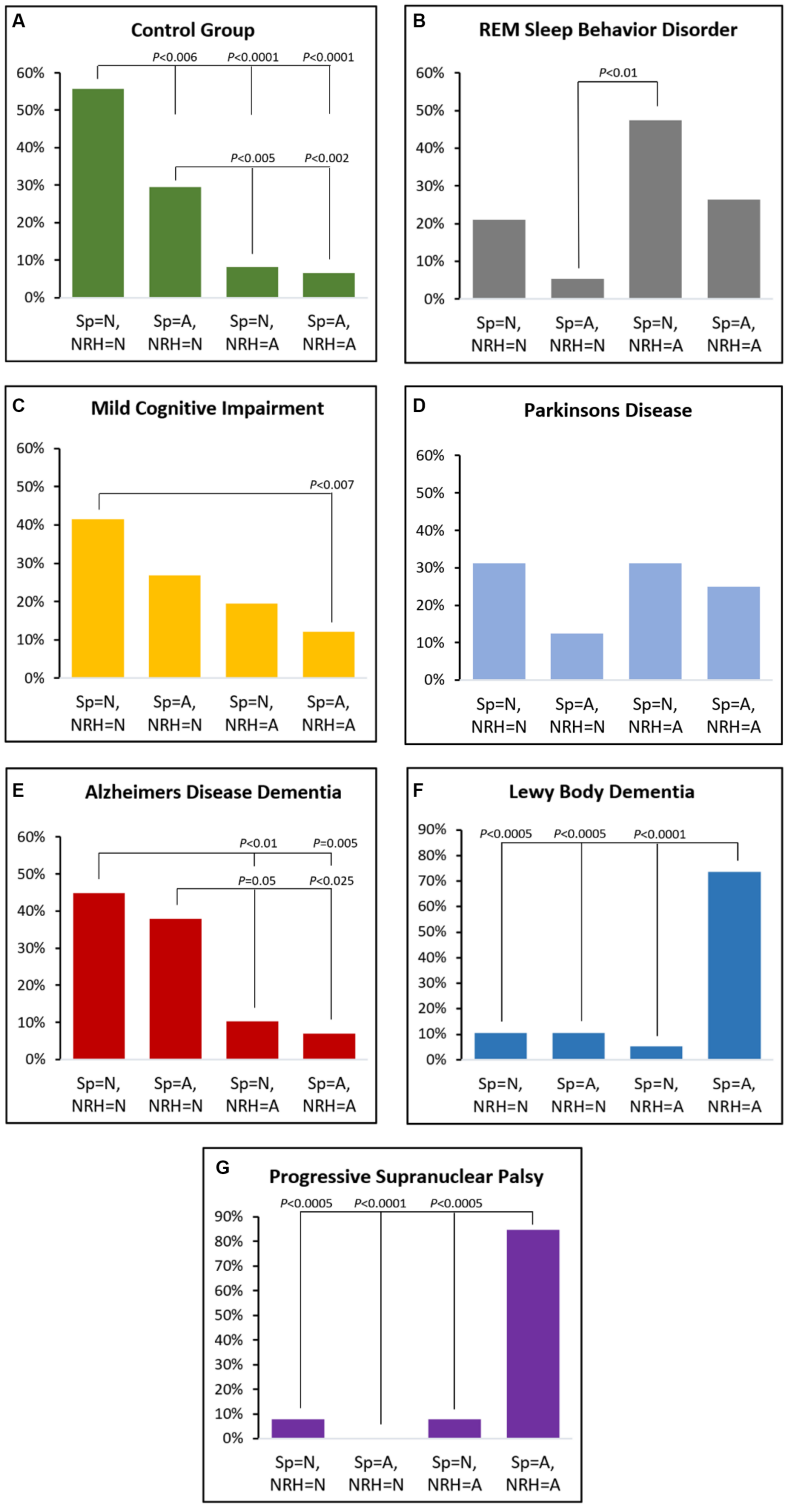
Proportional distributions by group across the four orthogonal categories based on normal (N) or abnormal (A) by spindle duration (Sp) or non-REM hypertonia (NRH).

Within-group distributions across the four biomarker combinations are presented in Figure 2. The proportion of the CG group with normal spindle duration and normal NRH (56%) was greater than the other orthogonal combinations (*p* < 0.006, all OR > 3, 95% CI range: 1.4–40.1). CG participants having abnormal spindle duration and normal NRH (30%) were also greater than those with abnormal NRH and normal spindle duration (8%) or abnormal spindle duration (7%) (*p* < 0.005; both OR > 4.7, 95% range: 1.6–18.9).

MCI patients were more likely to have normal spindle duration with normal NRH (42%) versus abnormal spindle duration and abnormal NRH (12%) (*p* < 0.007; OR = 5.1, 95% CI: 1.7–15.7).

The proportion of iRBD participants with normal spindle duration and abnormal NRH (47%) was greater than those with abnormal spindle duration and normal NRH (5%) (*p* < 0.01; OR = 16.2, 95% CI: 1.8–147.1).

In ADem the frequency of combined normal spindle duration and normal NRH (45%) was greater than those with abnormal NRH and either normal spindle duration (10%) or abnormal spindle duration (7%) (*p* < 0.01; both OR > 7.0, 95% CI range: 1.7–28.6). The proportion with abnormal spindle duration with normal NRH (38%) was also greater than either orthogonal category having abnormal NRH (*p* < 0.05; both OR > 5.3, 95% CI range: 1.3–41.7).

The proportion of LBD with abnormal spindle duration and abnormal NRH (74%) was greater than all other orthogonal combinations (*p* < 0.0005; all OR > 23.8, 95% CI range: 4.0–481.9) except the PSP group, which exhibited an even greater frequency of abnormal spindle duration and abnormal NRH (85%) (all *p* < 0.0005; all OR > 66, 95% CI range: 5.2–833.6).

### 3.7. Across group distributions of NREM biomarker combinations

**Figure 3:**
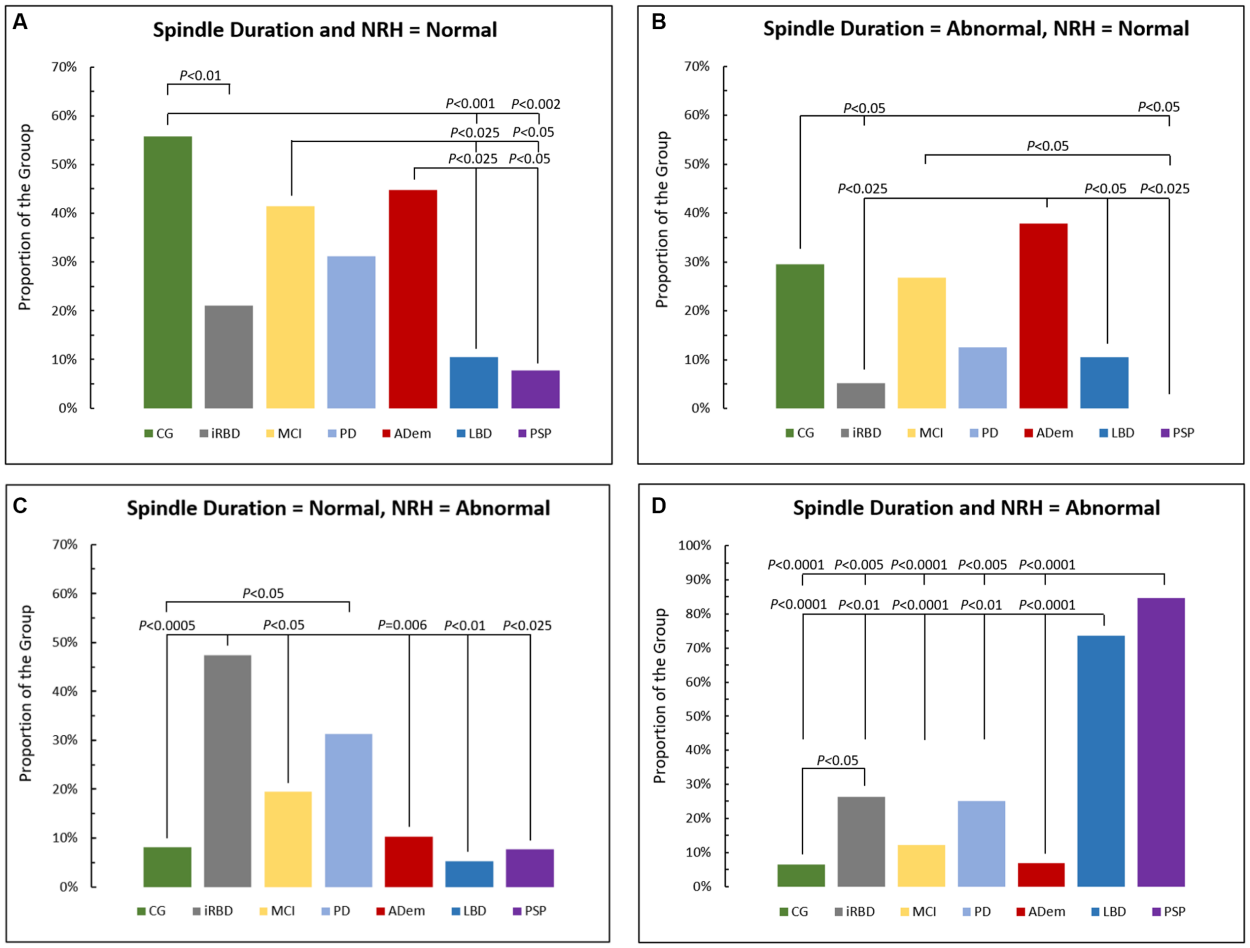
Comparison of proportions of cases across groups with **(A)** normal spindle duration and Non-REM hypertonia (NRH), **(B)** abnormal spindle duration and normal NRH, **(C)** normal spindle duration and abnormal NRH, and **(D)** abnormal spindle duration and NRH. CG, control group; iRBD, isolate REM sleep behavior disorder; MCI, mild cognitive impairment; PD, Parkinson disease; AD, Alzheimer’s disease dementia; LBB, Lewy body dementia; PSP, progressive supranuclear palsy.

The combination of normal spindle duration and normal NRH presented in Figure 3A was greater in CG (56%), MCI (42%), and ADem and versus LBD (11%) and PSP (8%) (*p* < 0.05), and when CG was compared to iRBD (21%) (*p* < 0.01).

Figure 3B displays relative distributions for abnormal spindle duration and normal NRH with ADem (38%) greater than iRBD (5%), LBD (11%) and PSP (0%), while CG (30%) was greater than iRBD, and both CG and MCI (27%) were greater than PSP (all *p* < 0.05).

Figure 3C shows the combination of normal spindle duration and abnormal NRH, with iRBD (47%) greater versus CG (8%), MCI (20%), ADem (10%), LBD (5%), and PSP (8%), and when PD (31%) was compared to CG (*p* < 0.05).

Those with both abnormal spindle duration and abnormal NRH (Figure 3D) were predominantly seen in PSP (85%) and LBD (74%), significantly more than in CG (7%), iRBD (26%), MCI (12%), PD (25%), and ADem (7%) (all *p* < 0.01), and more often in iRBD when compared to CG (*p* < 0.05).

## 4. Discussion

This proof-of-concept study examined an approach for distinguishing neurodegenerative disorders based on application of novel non-REM sleep biomarkers, both individually and combined. Unlike non-REM sleep stages N1, N2 and N3, both spindle duration and NRH distinguished across-group differences and identified unique within-group patterns. This novel approach could have future widespread applicability given the capacity for in-home acquisition by the SP, which included 90% of study participants in this study, and use of automated detection of biomarker severity scores that exhibited strong between-night reliability and uniquely characterized a diverse range of neurodegenerative disorder types.

Sleep spindles appear to originate in the thalamus and provide an index of connectivity of the hippocampo-cortical circuitry involved with memory consolidation (21). The spindle detection algorithms used in this study were triggered by recognition of peaks in sigma power, included power bands that captured both fast and slow spindles, and were limited in length, i.e., patterns consistent with other studies (22, 23). Abnormal spindle duration was predominant in those with dementia associated with presumed accumulations of α-synuclein or 4-R tau pathology, and to a lesser degree AD-related synaptic loss. Despite reduced sleep times, iRBD patients exhibited similar spindle duration as the CG, probably due to age-related group differences.

Topographical mapping of spindle sigma power is typically central in origin, however spindle detection from frontal region demonstrated near perfect night-to-night reliability which could be expected from a genetic-like biomarker (24) and across group delineations consistent with neurodegeneration (8, 21–23, 25). We found spindle duration and spindle density were essentially equivalent in recognizing group differences. While spindle duration was selected for the majority of the analyses, additional investigations are needed to determine the conditions under which specific spindle measures are most useful as prodromal biomarkers for dementia, especially in patients with shorter disease duration or compromised sleep architecture.

The proportion of ADem patients who exhibited abnormal spindle duration was no greater than the CG, MCI, PD and iRBD groups, suggesting that clinical and/or imaging disease-specific ADem severity measures, or perhaps other novel spindle characteristic properties (i.e., amplitude, average spindle duration, fast versus slow spindle ratios, spindle-slow oscillation coupling properties, regional changes in topographic spindle expression, etc.) may be needed to delineate the relationship between sleep spindles and cognitive decline in ADem (23, 26, 27). Longitudinal studies are currently underway to expand the number of sleep records in each group while concurrently acquiring objective daytime measures of memory consolidation in an effort to evaluate the potential usefulness of spindle duration as a biomarker to predict age-related cognitive decline and to explore the relationships between the various sleep spindle measures (e.g., fast versus slow, occurring in stage N2 versus N3, duration versus density, etc.) across neurodegenerative disorder groups (22, 23, 25).

The pathophysiology underlying NRH remains unclear, however one hypothesis is that NRH reflects degeneration in GABAergic neurons in the substantia nigra and occurs primarily in those with synucleinopathies and PSP (28, 29). Thresholds applied to EMG power for detection of NRH were designed to ensure events were not called as a result of abnormal slowing in LBD patients who are awake (15). It appears that NRH may have utility in assessing advanced stages of synucleinopathies when REM sleep is limited, or detection impaired by delta and theta intrusion. Data are currently being acquired to further investigate the association between NRH and RSWA across a range of Parkinsonian spectrum disorders (20).

Abnormal spindle duration coupled with abnormal NRH may suggest a pattern of neurodegeneration shared by different Parkinsonian spectrum disorders, given it was predominant in LBD and PSP patients but present in only 7% of ADem patients. Longitudinal studies are needed to determine whether the 26% of iRBD patients having both of these non-REM sleep abnormalities are at higher risk of eventual phenoconversion than the 47% of iRBD patients with abnormal NRH coupled with normal spindle duration, and whether these markers may predict a predominant motor (i.e., prodromal PD) or cognitive impairment (prodromal DLB, or concomitant RBD with ADem) trajectory in suspected synucleinopathy populations.

As compared to the other subgroups, the PD cohort exhibited the most homogeneous distributions across the orthogonal categories. Thirty-one percent exhibited the pattern most common in CG (i.e., normal spindle duration and NRH), 31% exhibited a pattern most common in the iRBD group (i.e., normal spindle duration and abnormal NRH), and 25% exhibited the pattern typical of LBD with abnormalities in both spindle duration and NRH biomarkers. Future studies in patients with well-documented symptomatology will be needed to clarify the role of NREM sleep biomarkers in monitoring Parkinson progression.

Previously we reported abnormal NRH was associated with Parkinsonian spectrum disorders (i.e., PD, LBD and PSP) independent of SSRI/SNRI use (15). In this study, SSRI/SNRI use was associated with abnormal NRH, but with a potential interaction with diagnostic group that would likely achieve statistical significance with slightly larger group sample sizes. Studies are currently underway to expand the data sets for MCI, PD, iRBD, ADem, and LBD groups in an effort to clarify the relationship between SSRI/SNRI use and NRH (i.e., causal or coincidental) and to compare the influence of SSRI/SNRI use on both RSWA and NRH.

This study has several limitations. It provided preliminary evidence to suggest unique patterns can be elucidated in neurodegenerative disorder groups when clinical cutoffs were selected, and combinations of normal/abnormal sleep biomarkers were combined. These findings, however, were based on relatively small data sets, the presumption that patients were accurately assigned into each neurodegenerative disorder group, and uncertainty as to disease progression, i.e., early vs. late stage.

The extraction of sleep biomarkers from a frontal montage enabled self-application and in-home acquisition but deviated from conventional in-laboratory montages (16). Sleep staging is more difficult in patients with a NDD with any montage, however we previously demonstrated that auto-staging from a frontal montage combined with technical review was superior to visual staging of PSG recordings using the standard montage for detection of stages N3 and REM in iRBD patients (20). Importantly, the automated NRH and spindle duration algorithms required no technical review to achieve the reported detection accuracy and reliability.

This study was also limited by inconsistent across-site acquisition of neuropsychological testing, which precluded exploration of interrelationships between the sleep biomarker severities and the specific type and severity of cognitive decline. Further, the results need to be externally validated, given that the clinical cutoffs were both selected and then applied within the same data set. New data sets and longitudinal recording are being acquired to independently validate the repeatability and generalizability of these findings.

In summary, these preliminary findings suggest that NREM sleep biomarkers may aid in the discrimination of different neurodegenerative disorders. Larger prospective cohort studies will be needed to validate these proposed orthogonal classifications of sleep biomarkers.

## Data availability statement

The data supporting the conclusions of this article will be made available by the authors, without undue reservation.

## Ethics statement

The studies involving humans were approved by Chesapeake Research IRB, Mayo Clinic IRB, VA Puget Sound IRB, Banner Healthcare System IRB, UCSF IRB, and Mass General Hospital IRB. The studies were conducted in accordance with the local legislation and institutional requirements. The participants provided their written informed consent to participate in this study.

## Author contributions

DL: Conceptualization, Formal analysis, Methodology, Writing – original draft. TN: Conceptualization, Resources, Supervision, Writing – review & editing. CW: Conceptualization, Project administration, Resources, Writing – review & editing. DT: Project administration, Resources, Writing – review & editing. DS: Resources, Supervision, Writing – review & editing. JH: Resources, Supervision, Writing – review & editing. JL-I: Project administration, Resources, Writing – review & editing. CB: Funding acquisition, Project administration, Writing – review & editing. GM: Conceptualization, Writing – review & editing. BB: Funding acquisition, Methodology, Writing – review & editing. ES: Conceptualization, Methodology, Resources, Writing – original draft.
